# Performance Parameters of Inductively Coupled Plasma Optical Emission Spectrometry and Graphite Furnace Atomic Absorption Spectrometry Techniques for Pd and Pt Determination in Automotive Catalysts

**DOI:** 10.3390/ma13225136

**Published:** 2020-11-14

**Authors:** Marin Senila, Oana Cadar, Lacrimioara Senila, Sarah Böringer, Karine Seaudeau-Pirouley, Andrea Ruiu, Patrick Lacroix-Desmazes

**Affiliations:** 1 National Institute for Research and Development of Optoelectronics Bucharest, Research Institute for Analytical Instrumentation, 400293 Cluj-Napoca, Romania; oana.cadar@icia.ro (O.C.); lacri.senila@icia.ro (L.S.); 2 Fraunhofer Institute for Chemical Technology ICT, Department Environmental Engineering, Group Reaction and Separation Techniques, 76327 Pfinztal, Germany; Sarah.Boeringer@ict.fraunhofer.de; 3 Innovation Fluides Supercritiques (IFS), Bâtiment INEED, BP16109, 26300 Alixan, France; k.seaudeau@supercriticalfluid.org; 4 Institut Charles Gerhardt Montpellier (ICGM), Univ Montpellier, CNRS, ENSCM, 34095 Montpellier, France; andrea1.ruiu@gmail.com

**Keywords:** palladium, platinum, ICP-OES, catalysts, method validation, microwave digestion

## Abstract

Palladium (Pd) and platinum (Pt) are extensively used as catalysts in the petrochemical and automotive industries, and due to high demand for them on the market, their recycling from spent supported catalysts is clearly needed. To assess the content of Pd and Pt in catalysts in order to establish their commercial value or to evaluate the recovery efficiency of technologies used for recycling, reliable analytical methods for determination of these elements are required. Spectrometric methods, such as inductively coupled plasma optical emission spectrometry (ICP-OES) and graphite furnace atomic absorption spectrometry (GFAAS) are powerful tools that can be employed for the determination of Pd and Pt in various sample matrices. However, these methods allow only the injection of liquid samples. In this regard, the digestion of solid sample by microwave-assisted acid extraction procedures at high pressures and temperatures is often used. In this study, a microwave acid digestion method was optimized for the extraction of Pd and Pt from spent catalysts, using a four-step program, at a maximum 200 °C. The resulting solutions were analyzed using ICP-OES, at two different wavelengths for each metal (Pd at 340.458 and 363.470 nm, and Pt at 265.945 and 214.423 nm, respectively) and using GFAAS (Pd at 247.64 nm, Pt at 265.94 nm). Five types of spent catalyst were analyzed and the standard deviations of repeatability for five parallel samples were less than predicted relative standard deviations (PRSD%) calculated using Horvitz’s equation for all the analyzed samples.

## 1. Introduction

The six platinum group metals (PGMs) (platinum, palladium, ruthenium, rhodium, osmium, and iridium) offer specific physicochemical properties, having high stability, resistance to chemical agents, malleability, and being used in a variety of industries [1,2,3]. The primary resources for PGMs are generally very scarce; only several countries in the world have quarries with these elements, largest quarries being located in South Africa, Russia, Canada, Zimbabwe, and United States of America [4]. On the other hand, the consumption of PGMs continuously increased in recent years (e.g., from 90 kt in 2010 to 106 kt in 2015) [5]. Palladium (Pd), platinum (Pt) and rhodium (Rh) are extensively used in the automotive industry as catalysts to reduce concentration levels of harmful compounds (CO, NO_x_, hydrocarbons) in exhaust emissions [6]. In catalysts, these elements are dispersed on a substrate (carrier) with porous structure (ceramic or metallic), to ensure their catalytic performances [7,8,9].

Modern and highly efficient automotive catalysts should be operative for a maximum of 80,000–90,000 km [10]. In reality, they are used for a longer distances even if their efficiency decreases as a result of exposure to severe conditions (loss of metals used for catalytic reaction, changes in the distribution of the metals in the catalytic support, and accumulation of impurities such as organic compounds or water) [9]. As a result, important amounts of waste catalysts containing Pd and Pt are continuously produced.

The high value of the precious metals and the high demand on the market, correlated with prevention of natural resources exhaustion, lead to a clear need for recycling of Pd and Pt from used catalyst. This also ensures the lowering of the environmental pollution, linked both to the PGMs’ extraction and catalysts’ disposal. According to the literature, the concentrations of Pd and Pt in catalysts is relatively low, below 1000 ppm for Pd and 2000 ppm for Pt, respectively [6,9].

To recover Pt and Pd from used catalysts, extraction technologies classically based on pyrometallurgical and hydrometallurgical processes are used [2,3,10,11]. To improve efficiency of hydrometallurgical process, microwave-assisted leaching and cloud point extraction were also used [12]. Alternatively, green methods based on extraction using chelating agents were developed [13,14]. The efficient recovery and purification of Pd and Pt from spent catalyst is economically desirable. In order to evaluate the amounts of these elements in used catalysts and to assess the recovery efficiency of the extraction technologies, reliable and precise analytical methods for their determination are required. The direct determination of precious elements in solid catalysts is possible by X-ray fluorescence (XRF), with the main advantage of avoiding sample preparation. However, in the case of quantitative analysis, this technique should be carefully used due to the heterogeneity of the catalyst sample and the flatness of their surface, which can affect the results [15]. For the majority of other spectrometric determination techniques, a preliminary stage of sample preparation to extract the analyte in liquid solution is required. In this approach, the resulted liquid solution is homogeneous and it allows the determination of the concentration of metals by different spectrometric instruments that are available in analytical laboratories. The preliminary step of sample preparation is extremely important in obtaining reliable results since the quantitative recovery of the analyte and also the absence of contamination are essential.

There are several ways of sample preparation, the most used: leaching in a mixture of acids (HCl, HNO_3_, HF, H_2_SO_4_) [16,17,18,19], fire assay [6,9] and fusion with different chemicals [9,20]. The sample preparation procedure is chosen to ensure as much as possible the quantitative extraction of analyte in solution. Some other factors such as costs of digestion devices, time consumption, amount of necessary sample amount, energy and reagent consumption should also be considered. A fire assay is a digestion and preconcentration procedure recognized for providing high recoveries for precious metals. It consists in sample melting in a furnace at a temperature over 1000 °C in a flux of chemicals, in the presence of a collector (usually Pb, Cu, NiS) and a reducing agent. Melting is followed by cupellation and subsequently by digestion of metals in acids (usually HCl and NHO_3_) [16]. In this procedure, the metals are collected and concentrated from a large sample (10–50 g), which is an advantage when considering preconcentration. However, this large amount of sample required may represent also a limitation if not sufficient sample amount is available. Fusion is another digestion procedure that uses alkaline hydroxides and an alkali metal peroxide or nitrate in a crucible to melt sample at a high temperature (up to 1000 °C) for about 30 min. After cooling down, the melt is subsequently dissolved in a diluted mineral acid to extract the precious metals [20].

The direct leaching of metals from the sample in liquid solution using different mineral acids is desirable in order to avoid the multistep procedures based on melting, which are time consuming, require specific devices for melting, involve higher energy consumption and use specific chemicals in melting processes. Microwave-assisted extraction is based on the use of the microwave energy to transfer the analytes from the matrix into the extraction solution, in this case a mixture of acids. Microwave-assisted extraction leads to increased recovery rates, reduced extraction time and reduction of reagent consumption [21].

Several analytical spectrometric techniques are applied for the determination of Pt and Pd in solid samples such as spectrophotometry [22], graphite furnace atomic absorption spectrometry (GFAAS) [22,23,24] and inductively coupled plasma optical emission spectrometry (ICP-OES) [7,16,17,25,26]. Among them, ICP-OES is considered one of the most appropriate instrumental techniques for determination of Pd and Pt in different samples due to its multi-element capability, wide linear dynamic range and good precision. GFAAS is a single element technique, being time-consuming compared to ICP-OES, but has the advantage of lower limits of detection and good selectivity [27]. Previous studies reported LODs for determination of PGMs in the ranges of sub ppb to ppb for GFAAS and ppb to tens of ppb for ICP-OES [5].

Method validation is a critical key for a laboratory in order to produce consistent analytical results. When the performance of a method is evaluated, several factors should be considered: use of certified reference materials; comparison of results obtained from different methods or different laboratories; methodical assessment of the factors that influence the result; evaluation of the measurement uncertainty considering the theoretical principles and practical experience [28,29].

Analytical laboratories shall apply procedures for estimating uncertainty of measurements, and there are several options to estimate the uncertainty, as found in the literature [30]. The conventional model for measurement uncertainty assessment is based on the identification and estimation of individual components of uncertainty from the measurement procedure, that are subsequently combined as standard uncertainties in order to obtain the combined standard uncertainty. This approach allows the identification of the critical control points, but on the other hand this is time consuming and involves an extensive knowledge of the analytical procedure. An easier way of uncertainty estimation is the in-house validation that involves the determination of the method performance parameters [30,31].

Despite the existing data published in literature regarding Pd and Pt quantification in catalyst samples, there is a lack of information on performance parameters of methods for a full validation of the process. Also, a comparison of analytical performances of two different spectrometric methods that can be employed for Pd and Pt determination is missing.

Even if microwave-assisted acid extraction is attractive in terms of simplicity, speed in analysis, low consumption of chemicals and energy, the recovery of this procedure should be carefully evaluated. In case of some type of samples and acids used for digestion, the recovery may be not quantitative. As an example, for ore samples, were reported recoveries of only 46–55% for Pt and 61–88% for Pd, when *aqua regia* and microwave heating followed by ICP-MS determination was applied [16].

One of the aims of this study was to evaluate the recovery rate and to optimize the HNO_3_:HCl ratio used for the microwave-assisted acid extraction of Pd and Pt from spent catalysts using a certified reference material with an appropriate matrix. Another aim was to compare the analytical performances of ICP-OES and GFAAS techniques for determination of Pd and amounts in the supported catalysts. The methods were characterized in terms of selectivity, limit of detection (LOD), limit of quantification (LOQ), linearity of calibration curve, precision, recovery and uncertainty. The assessment of uncertainty was obtained using in-house validation data. Finally, the two validated methods were applied for Pd and Pt determinations in spent catalysts samples.

## 2. Materials and Methods

### 2.1. Reagents, Standard Solutions and Certified Reference Material

Nitric acid 65% (*w*/*w*) analytical grade, hydrochloric acid 37% (*w*/*w*) analytical grade, single-element standard solutions 1000 mg L^−1^ of Pd, Pt, Al, Fe, Mg and Si were purchased from Merck (Darmstadt, Germany). Ultrapure water (18 MΩ cm) prepared with Milli-Q system Direct Q3 (Millipore, Molsheim, France) was used throughout the experiments.

A certified reference material (CRM), NIST SRM 2557—pulverized recycled monolith (National Institute of Standards and Technology, Gaithersburg, MD, USA), was analyzed to validate the determinations by ICP-OES and GFAAS.

Spent catalysts were provided by Heraeus Deutschland GmbH and Co. KG (Hanau, Germany).

### 2.2. Methods and Instrumentation 

The spent supported catalyst samples were ground in a mortar grinder and sieved, to obtain a fine powder with particle size below 100 µm mesh sieve. Amounts of 0.500 g catalyst sample was mixed with 2 mL of nitric acid 65% (*w*/*w*) and 6 mL/8 mL/10 mL/12 mL or 15 mL hydrochloric acid 37% (*w*/*w*), in PTFE digestion vessels of Speedwave Xpert Berghof microwave digester (Berghof, Germany). Then, the digestion program of the microwave system using a four-step program of heating at 100 °C, 200 °C, then cooling at 100 °C, and 25 °C in a total time of digestion of 40 min was applied. The insoluble part was filtered through 0.45 µm Whatman cellulose membrane filters (Amersham, UK) and the filtrate was collected in a volumetric flask and diluted up to 100 mL with ultrapure water. Reagent blank containing only acids and ultrapure water, without sample, was prepared using the same microwave-assisted digestion procedure.

The content of Pd in the obtained solutions was measured by ICP-OES (Optima 5300DV, Perkin Elmer, Norwalk, CT, USA) or GFAAS (PinAAcle 900T Perkin Elmer, Norwalk, CT, USA), under the instrumental conditions presented in Table 1. Eight-points external calibration was used for the calibration of ICP-OES, using the reagent blank and 7 calibration standard solutions of 0.10, 0.50, 2.0, 5.0, 10, 15, 20 mg L^−1^ prepared by appropriate dilutions of single-element standard solutions 1000 mg L^−1^ of Pd or Pt with the reagent blank solution (prepared from 20 mL NHO_3_ 65% (*w*/*w*) and 120 mL HCl 37% (*w*/*w*) diluted to 1000 mL with ultrapure water in order to assure the similar concentration of acids in samples and in calibration standards).

In the case of Pd measured by GFAAS, 7-points external calibration was plotted using the reagent blank and 6 calibration standard solutions of 10, 20, 40, 60, 80, 100 µg L^−1^. In the case of Pt measured by GFAAS, the reagent blank and 6 calibration standard solutions of 20, 30, 40, 60, 80, 100 µg L^−1^ were used. In this technique, the calibration standards were prepared by auto dilution of the highest concentrated standard solution (100 µg L^−1^ of Pd and Pt, respectively) with the reagent blank using the instrument autosampler.

In order to check the calibrations, the highest concentrated standard solution from each calibration curve was measured to be in the range of ±10% from the theoretical value, and in all cases the measured value was in the required range. For an independent check of calibrations in ICP-OES, a solution of 4.0 ± 0.4 mg L^−1^ Pd and Pt prepared independently form the calibrations standards was used, and the measured concentrations were 4.11 mg L^−1^ for Pd and 3.98 mg L^−1^ for Pt. For GFAAS, a solution of 50 ± 5.0 µg L^−1^ Pt and Pd prepared by dilutions was measured as a sample, and the measured concentrations were 49 mg L^−1^ for Pd and 48 mg L^−1^ for Pt, which are satisfactory results.

Total carbon (TC) was measured using a total carbon analyser Multi N/C 2011S (Analytic Jena, Germany) using the module for solid samples. Amounts of about 10 mg of catalysts samples were weighted in porcelain nacelles, introduced in analyser oven at 1200 °C in an oxygen stream, where C is oxidized to CO_2_, then transported and measured by an infrared (IR) detector.

### 2.3. Strategy for Methods’ Validation

The two spectrometric methods were characterized regarding selectivity, LOD, LOQ, linearity of calibration curves, precision, recovery and measurement uncertainty.

Selectivity is defined as the ability of a method to correctly quantify the analytes in the presence of interferences, and in case of spectrometric methods, this is related to possible interferences at working wavelengths of analyte. To evaluate this parameter, for ICP-OES, a solution of 1 mg L^−1^ Pd and Pt, also containing 100 mg L^−1^ Al, Si, Mg and Fe was introduced into ICP-OES and the emission wavelengths of 340.458 and 363.470 nm for Pd, and 265.945 and 214.423 nm for Pt were checked to observe if spectral interferences will appear. Also, the standard addition approach was used for both ICP-OES and GFAAS. The analyte was measured in a test sample of extraction solution of catalyst, then selectivity was evaluated by recovery of a spike of 2 mg L^−1^ Pd and Pt in ICP-OES, and 20 μg L^−1^ Pd and Pt in GFAAS. The self-imposed target was spike recovery of 100 ± 10% for the two spectrometric methods.

LOD was calculated using the three times standard deviation of background assessed from 10 measurements of reagent blank containing 2 mL NHO_3_ 65% (*w*/*w*) and 12 mL HCl 37% (*w*/*w*) diluted to 100 mL with ultrapure water (3σ criterion) [30]. LOQ was considered as 3 × LOD. Values of LOD and LOQ in solid sample were calculated considering the sample digestion protocol.

Evaluation of the linear calibration function was made according to ISO 8466-1 [32], by studying homogeneity of dispersion at the limits of the calibration ranges. Homogeneity of dispersion was appraised as variance ratio at the limits of working ranges (PG) calculated so that the PG value is higher than 1:

(1) $$PG=\frac{s_{1}^{2}}{s_{2}^{2}}\;or\;PG=\frac{s_{2}^{2}}{s_{1}^{2}}$$

where $s_{1}^{2}$ and $s_{2}^{2}$ are standard deviations of measurements corresponding to the lowest and the highest concentrations of the standards from the calibration curves.

*PG* value is then compared with the critical value of Fisher–Snedecor distribution (F_9;9;0.99_ = 5.35). If *PG* < F_9;9;0.99_, the difference between variances is not significant and the working range is considered acceptable. One other target for linearity study was to achieve correlation coefficients ≥0.995, usually considered satisfactory for linear calibration.

Precision of an analytical method is given by the random errors and is evaluated as repeatability and reproducibility. In this study, repeatability on the same equipment, and reproducibility using the two different techniques (ICP-OES and GFAAS) were considered. The accuracy of the two spectrometric methods was assessed through a recovery study by analysing NIST SRM 2557—pulverized recycled monolith CRM.

The measurement uncertainty was assessed based on the in-house validation process, considering that it comprises the whole analytical procedure. In this way, it was assumed that the main parameters affecting the measurement uncertainty of the analytical result are clustered into two main components: accuracy and precision of the method [30]. The combined standard uncertainty (*uc*) is then calculated by using the bias from the certified value of CRM and standard deviation from accuracy study:

(2) $$u_{c}=\sqrt{B^{2}+u(C_{R)}{}^{2}}$$

where *B* is bias obtained in accuracy study in CRM analysis and *u(C_R_)* is the standard deviation obtained in precision study in CRM analysis.

Using the combined standard uncertainty, the expanded uncertainty (*U*) was calculated considering a cover factor *k* = 2, for a level of confidence of 95%.

(3) $$U=ku_{c}$$

## 3. Results and Discussion

### 3.1. Optimization of Microwave-Assissted Acid Wet Digestion Stage

**Figure 1:**
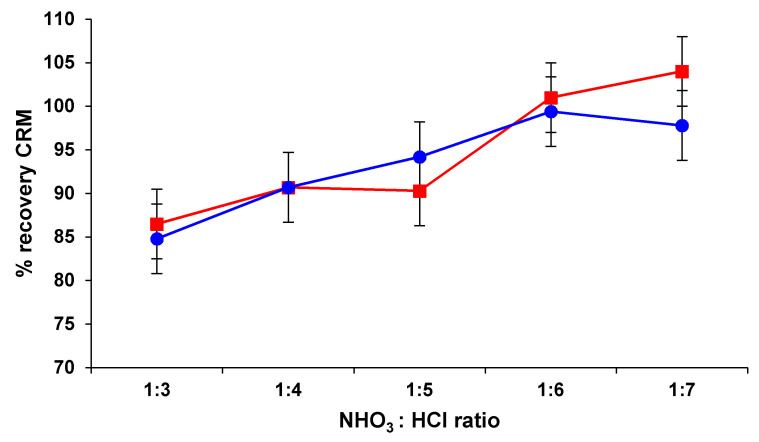
Influence of HCl and HNO_3_ ratios on Pd (red color) and Pt (blue color) recovery degree (%) from catalyst certified reference material (CRM) NIST SRM 2557.

The quantitative recovery of the analytes on the extraction stages of the analytical protocols is desired in order to provide reliable results. The wet acid digestion of precious metals is a simple, rapid and inexpensive procedure. The recovery efficiency of acid extraction strongly depends on the chemical solubility of analytes, their concentrations and type of matrix. PGMs are resistant to single mineral acids, thus mixtures of HCl, HNO_3_, HClO_4_, and HF are usually applied for digestion. Pd and Pt are soluble in a mixture of HCl and HNO_3_, but depending on the solid matrix these can be non-quantitatively extracted. Insufficient amount of acids, incomplete wetting of solid samples, and occlusion of metals in the solid support can lead to an incomplete digestion. Microwave heating at high temperature and pressure can significantly improve the leaching. The ratio of mineral acids used for leaching may influence the extraction. In order to evaluate this factor for the digestion of 0.500 g of sample, mixtures of concentrated HNO_3_ and HCl in ratios of 1:3 (*v*/*v*), 1:4(*v*/*v*), 1:5 (*v*/*v*), 1:6 (*v*/*v*), and 1:7 (*v*/*v*) were used in microwave conditions, by applying the same digestion program. The CRM NIST SRM 2557 having known content of Pd and Pt was used to assess the recovery. Pd and Pt concentrations in resulting solutions were measured by ICP-OES. Three replicates were carried-out for this experiment, with an average uncertainty expressed as standard deviation of repeatability of 5%. The results are presented in Figure 1.

The recovery of Pd ranged between 86.5–104%, generally increasing from the ratio HNO_3_:HCl of 1:3 (*v*/*v*) to the ratio of 1:6 (*v*/*v*), with no significant change if the ratio increased to 1:7 (*v*/*v*). In the case of Pt, this trend of leachability was almost similar: the highest recovery of 99.4% was found at a ratio HNO_3_:HCl of 1:6 (*v*/*v*), with no significant variation at the ratio of 1:7 (*v*/*v*). Accordingly, it was considered that for the quantitative leaching of Pd and Pt from 0.500 g of catalyst sample, the use 2 mL of HNO_3_ 65% (*w*/*w*) and 12 mL HCl 37% (*w*/*w*) in microwave conditions represents an appropriate digestion method.

The developed microwave-assisted extraction method has a total time of 40 min, i.e., much shorter compared to the fire assay and fusion procedures as presented in other works [6,9,16]. Also, it requires only a few mL of HNO_3_ and HCl and only 0.500 g of sample are needed. Its disadvantage consists in the lack of total sample dissolution, part of the support catalysts remaining as a solid residue that is filtered whereas in the other two methods the whole sample is melted and precious metals are quantitatively extracted. For some samples, this may lead to low recoveries, but in the case of supported catalyst samples, due to the metals deposition at the surface of the support and also due to the support porosity, it was found that good recoveries were achieved.

### 3.2. Selectivity

In the spectrometric determinations of trace elements from samples with complex matrices (in this case, the catalyst support), spectral and non-spectral interferences can be produced by the high concentrations of other elements resulting from digestion of the support. Solutions resulting from acid digestion of catalyst samples can usually contain high amounts of Al, Si and Fe, which may cause interferences. For ICP-OES, selectivity was checked by observing the emission wavelengths at two different wavelengths for each metal (Pd—340.458 and 363.470 nm, and Pt—265.945 and 214.423 nm, respectively). As no spectral interferences were observed, all the selected working wavelengths were considered free of spectral interferences for determination of Pd and Pt from catalyst samples.

Selectivity was also assessed by recovery of a spike in the original samples of 2 mg L^−1^ element in ICP-OES and 20 μg L^−1^ element in GFAAS. The recoveries of spikes in this case were within 90–110% for the two elements and techniques.

### 3.3. Limit of Detection, Quantification and Linearity

Generally, LOD is considered as the lowest concentration that can be measured consistently. There are several methods for the estimation of LOD, but the most widely used and appropriate method for the spectrometric techniques is to calculate this parameter as three times the standard deviation of the signal from the blank sample. Herein, LOD was calculated using the 3 times standard deviation of background assessed from 10 measurements of reagent blank. LOQ is the lowest concentration of analyte that can be measured with an acceptable level of accuracy and precision. In this paper, LOQ was calculated as 3 times the LOD. Values of LOD and LOQ were finally calculated in solid sample taking into account the sample digestion. The LODs ranged between 3.6–7.4 mg kg^−1^ (18–37 µg L^−1^ in liquid solution) element for ICP-OES technique, allowing thus quantification of concentrations higher than 10.8–22.2 mg kg^−1^ element (Table 2). Better LOQ was obtained for Pd at the wavelength of 340.458 nm (18 µg L^−1^) while for Pt the lowest LOQ was obtained at emission line of 265.945 nm (25 µg L^−1^). For this reason, the other performance parameters were assessed at the wavelengths of 340.458 nm for Pd and 265.945 nm for Pt, respectively. 

Our results for LOD in ICP-OES were in the range of those reported by Petrova et al. [26] of 7–57 µg L^−1^ for Pt and of 11–62 µg L^−1^ for Pd, in liquid solvents. Komendova [5] reported LOD in ICP-OES of 2 µg L^−1^ for Pd, and 20 µg L^−1^ for Pt, in liquid solution, while Gonzales Torres et al. [33] reported for Pt an LOD of 49 µg L^−1^. Nakajima et al. [25] reported a LOD for Pt in ICP-OES of 0.28 µg L^−1^, lower than in our study, but this was obtained after a separation and preconcentration step.

In GFAAS, LODs for Pd and Pt were 0.3 and 0.9 mg kg^−1^ (3.0 and 4.6 µg L^−1^ in liquid solution) and allowed determinations of concentrations higher than 1.8 and 2.7 mg kg^−1^ for Pd and Pt, respectively. Our LODs were higher than those reported by Jamali et al. [23] for Pd, of 0.02 µg L^−1^ after preconcentration, but in the same order of magnitude as those reported by Komendova [5] (0.5 µg L^−1^ for Pd and 4.5 µg L^−1^ for Pt in liquid solution), Eskina et al. [22] (1.8 mg kg^−1^ for Pd and 6.2 mg kg^−1^ for Pt is solid samples) or Bosch Ojeda et al. [34] (0.8 µg L^−1^ for Pd in liquid solution).

Variance ratio at the limits of working range and correlation coefficient calculated according to ISO 8466-1 [32] are presented in Table 2. Satisfactory linearity over the calibration range with correlation coefficients of 0.9964–0.9997 was obtained in the two methods, but it was superior in ICP-OES. The ratios s^2^_1/_s^2^_2_ calculated for measurements of lowest and highest concentrations from the calibration curves were below the critical value *F*_9;9;0.99_ = 5.35, indicating dispersion homogeneity and that the concentration range was satisfactorily chosen.

### 3.4. Precision and Accuracy

The precision study was accomplished in terms of repeatability and internal reproducibility using two different instruments (ICP-OES and GFAAS). Table 3 summarizes the results for the repeatability assay on 10 parallel CRM samples achieved on single equipment. Standard deviations of repeatability/limit of repeatability were then compared with predicted relative standard deviations (*PRSD*%) calculated according to Horvitz’s equation [35]:

(4) $$PRSD\%=2^{\left(1-0.5logC\right)}$$

where *C* is analyte concentration expressed as mass fraction in extracted liquid solutions.

As shown in Table 3, the relative standard deviations of repeatability (RSDr) were 2.92% and 4.19% for Pd and Pt, respectively in ICP-OES and 4.21% and 5.82% for Pd and Pt, respectively in GFAAS, lower than PRSD calculated for the concentration level of analytes in solutions of 6.6% for Pd and 7.5% for Pt, thus being considered as satisfactory. Faber and Brodzik [9] reported values for RSD of repeatability <5% calculated for Pd and Pt analyses in automotive catalysts by ICP-OES and X-Ray diffraction, similar to those found in our study.

To compare the results obtained by two different techniques, T test for dependent samples was used. No significant differences (at *p* < 0.05) were observed between datasets for the two methods (*p* = 0.74 for Pd, and *p* = 0.40 for Pt).

The accuracy of the methods was checked by analyzing a pulverized recycled monolith CRM NIST SRM 2557. The certified values of CRM, measured values and recovery degrees are presented in Table 4. For an instrumental analytical method, at a concentration of analyte of hundreds ppm, a recovery within the range 85–110% is considered to be satisfactory [36]. Recoveries over 100% may be caused by all the sources of uncertainty associated to the analytical procedure. The main sources of uncertainty for this method are: sample homogeneity, volumetric operations (volumetric flasks, pipettes used for sample and calibration standards preparation), purity of calibration standards, uncertainties of equipment (spectrometers and analytical balance), or possible contamination during sample preparation.The comparison of measurement results with the certified values was done by comparing the differences between the certified and measured values with the combined uncertainty of certified values and measured uncertainty [37].

The difference between average measured concentration (*C_m_*) and the certified values (*C_CRM_*) was calculated using Equation (5):

(5) $$\Delta_{m}=\left|C_{m}-C_{CRM}\right|$$

The combined uncertainty (*U_Δ_*), was calculated from the specified uncertainty (*U_CRM_*) and measured uncertainty (*U_m_*) expressed as standard deviation, using the formula:

(6) $$U_{\Delta }=\sqrt{U_{m}^{2}+U_{CRM}^{2}}$$

In case of ICP-OES, the difference between the certified and measured value of Pd was 6.0 mg kg^−1^, smaller than the expanded uncertainty (*k* × *U_Δ_*, *k* = 2) of 10.8 mg kg^−1^, while the difference between the certified and measured value of Pt was 20 mg kg^−1^, also smaller than the expanded uncertainty of 113 mg kg^−1^. In case of GFAAS, it was calculated for Pd a difference between the certified and measured value of 11.6 mg kg^−1^ and an expanded uncertainty of 23.4 mg kg^−1^, while for Pt the difference between the certified and measured value was 28 mg kg^−1^, and an expanded uncertainty of 99 mg kg^−1^. According to these values, there is no significant difference between the certified results and measured concentrations.

### 3.5. Measurement Uncertainty

The main sources of measurement uncertainty in an instrumental determination are: calibration reference materials, uncertainty of weighted reference solutions and samples, uncertainty of the calibration curve, and accuracy and repeatability of the method [38]. In this study, assuming that quality control included the total analytical procedure, thus two main components affect the uncertainty: bias obtained in accuracy study in CRM analysis and standard deviation obtained in CRM analysis. Using Equation (2), were calculated the combined uncertainties (*u_c_*), expanded uncertainties (*U*) for a cover factor *k* = 2 (P = 95%), and relative expanded uncertainties (*U_rel_*) for Pd and Pt measured by ICP-OES and FAAS, and the results are presented in Table 5.

The relative expanded uncertainties (*k* = 2, *P* = 95%) were of 6.4% and 10.7% in ICP-OES, and 14.9% and 10.3% in GFAAS, which are satisfactory parameters for a method that implies sample digestion.

### 3.6. Analysis of Spent Catalysts Samples

The results obtained in the analysis of five types of spent catalysts samples by microwave-assisted acid extraction and ICP-OES and GFAAS measurement, and their TC content are summarized in Table 6.

Total carbon (TC) was measured in order to evaluate the level of organic residues in spent catalysts. TC content varied in the range of 0.2–4.0%. The content of Pd was in the range of 0.0141–1.714%, while the content of Pt ranged between 0.0787–0.1998%. No significant differences between the results obtained by ICP-OES and GFAAS were obtained. 

## 4. Conclusions

The ICP-OES and GFAAS techniques were validated for the analysis of Pd and Pt in spent catalysts, demonstrating that both methods are suitable for the determination of these elements. The performance characteristics (LOD and LOQ, selectivity, linearity, accuracy, precision and measurement uncertainty) fulfilled the imposed targets. GFAAS provided better LOD, but ICP-OES had the advantage of wider linear ranges and speed of analysis. The methods were successfully applied for the analysis of catalysts samples, and no significant differences were observed between the results obtained by the two spectrometric techniques. A microwave-assisted acid extraction method was developed and optimized. An optimal ratio HNO_3_:HCl of 1:6 (*v*/*v*) with a total time of digestion of 40 min was found to give quantitative recoveries for Pd and Pt from supported catalysts samples. Compared with other digestion methods based on melting, this method is simpler, faster, involves fewer steps in sample handling, thus reducing sources of contamination, and requires fewer chemicals and amounts of samples. The LOQs obtained in ICP-OES allow the quantification of concentrations higher than 10.8 mg kg^−1^ for Pd and 15.0 kg^−1^ for Pt, while LOQs in GFAAS were 1.8 mg kg^−1^ for Pd and 2.7 kg^−1^ for Pt. To compare the results obtained by two different techniques, a *t*-test for dependent samples was used, and no significant differences (at *p* < 0.05) were observed between datasets for the two methods. The paper presents all the stages required to validate the method of Pd and Pt determination in catalysts using spectrometric techniques.

## Figures and Tables

**Table 1:** Operation conditions for Pd and Pt determination by inductively coupled plasma optical emission spectrometry (ICP-OES) and graphite furnace atomic absorption spectrometry (GFAAS).

ICP-OES	Parameters				
Plasma torch Background correction	Inductively coupled plasma, radial and axial viewing, RF power 1450 W Argon flow rates: Outer gas 12 L min^−1^; Intermediate gas 0.8 L min^−1^; Nebulizer gas 1 L min^−1^ Linear two points model, integration time 10 s and 5 successive measurements for each parallel sample				
Sample introduction	Spay chamber: cyclonic Nebulizer: Meinhard type C			Read delay: 60 s Peristaltic pump flow rate: 1.8 mL min^−1^	
Wavelengths	Pd 340.458 nm, axial viewing Pd 363.470 nm, axial viewing			Pt 265.945 nm, axial viewing Pt 214.423 nm, axial viewing	
**GFAAS**	**Parameters**				
Signal processing: Peak area; Read time: 5 s; Sample volume: 20 µL Background correction: Zeeman-effect					
Pd Wavelength—247.64 nm HCL current—20 mA			Pt Wavelength—265.94 nm HCL current—30 mA		
**Furnace Program Pd**					
Step	Temp (°C)	Ramp (s)		Hold (s)	Ar (mL min^−1^)
Drying	110	1		30	250
Drying	130	15		30	250
Ashing	900	10		20	250
Vaporization	2200	0		5	0
Cleaning	2450	1		3	250
**Furnace Program Pt**					
Step	Temp (°C)	Ramp (s)		Hold (s)	Ar (mL min^−1^)
Drying	110	1		30	250
Drying	130	15		30	250
Ashing	1300	10		20	250
Vaporization	2200	0		5	0
Cleaning	2450	1		3	250

**Table 2:** Working wavelengths, linear ranges, variance ratio at the limits of the calibration range (*PG*), correlation coefficients (*R*), limits of detection (*LODs*) and limits of quantification (*LOQs*) of the spectrometric methods used in the Pd and Pt analysis from catalysts.

Element, λ (nm)	Technique	Calibration Range	PG	R	LOD * (mg kg^−1^)	LOQ * (mg kg^−1^)
Pd 340.458 nm	ICP-OES	0.10–20 mgL^−1^	4.89	0.9997	3.6	10.8
Pd 363.470 nm	ICP-OES	0.10–20 mgL^−1^	3.56	0.9995	6.4	19.2
Pt 265.945 nm	ICP-OES	0.10–20 mgL^−1^	3.27	0.9994	5.0	15.0
Pt 214.423 nm	ICP-OES	0.10–20 mgL^−1^	5.05	0.9987	7.4	22.2
Pd 247.64 nm	GFAAS	10–100 µgL^−1^	4.12	0.9964	0.6	1.8
Pt 265.94 nm	GFAAS	20–100 µgL^−1^	2.49	0.9975	0.9	2.7

* LOD and LOQ with respect to the dissolved solid sample (amount of 0.5 g in 100 mL solution).

**Table 3:** Results obtained in the repeatability assay by ICP-OES and GFAAS in the Pd and Pt analysis of automotive spent catalysts (*n* = 5 parallel samples).

Measurements	ICP-OES		GFAAS	
	Pd	Pt	Pd	Pt
X_1_ (mg kg^−1^)	1825	828	1775	862
X_2_ (mg kg^−1^)	1756	808	1672	898
X_3_ (mg kg^−1^)	1850	790	1785	770
X_4_ (mg kg^−1^)	1813	876	1882	827
X_5_ (mg kg^−1^)	1722	799	1792	869
X_m_ (mg kg^−1^)	1793	820	1775	845
s (mg kg^−1^)	52	34	75	49
r (mg kg^−1^)	147	96	210	138
RSDr (%)	2.92	4.19	4.21	5.82

s—standard deviation, r—limit of repeatability (r = s × 2.8); RSDr—relative standard deviation of repeatability.

**Table 4:** Certified values of CRM, measured values (*n* = 5 parallel determinations) and the recoveries degree (%).

CRM	Technique	Certified Values ± U ^a^ (mg kg^−1^)		Measured Values ± U ^b^ (mg kg^−1^)		Recovery (%)	
		Pd	Pt	Pd	Pt	Pd	Pt
NIST SRM 2557	ICP-OES	233.2 ± 1.9	1131 ± 11	238.8 ± 5.3	1111 ± 56	102	98
	GFAAS			221.6 ± 5.3	1103 ± 49	97	95

^a^ U = expanded uncertainty (*k* = 2); ^b^ U = calculated expanded uncertainty (*k* = 2).

**Table 5:** Combined uncertainties, expanded uncertainties (*k* = 2, P = 95%), and relative expanded uncertainties for Pd and Pt measured by ICP-OES and GFAAS.

Uncertainties	ICP-OES		GFAAS	
	Pd	Pt	Pd	Pt
u_c_ (mg kg^−1^)	7.7	59.6	16.5	56.7
U (mg kg^−1^)	15.4	119	33.0	113
Associated mean concentration (mg kg^−1^)	238.8	1111	221.6	1103
Urel%	6.4	10.7	14.9	10.3

**Table 6:** Results obtained in the analysis of spent catalysts samples by ICP-OES, GFAAS, and total carbon (TC) analyser (mean ± *U* (*k* = 2), *n* = 5 parallel samples).

Sample	TC Analyser	ICP-OES		GFAAS	
	TC	Pd	Pt	Pd	Pt
	% (*w*/*w*)				
Catalyst 1	1.3 ± 0.1	0.4954 ± 0.0317	<0.0015	0.4932 ± 0.0735	<0.0003
Catalyst 2	4.0 ± 0.2	1.714 ± 0.1097	<0.0015	1.625 ± 0.2421	<0.0003
Catalyst 3	0.2 ± 0.1	<0.0011	0.1457 ± 0.0156	<0.0002	0.1398 ± 0.0144
Catalyst 4	2.1 ± 0.2	0.1790 ± 0.0115	0.0820 ± 0.0088	0.1758 ± 0.0262	0.0787 ± 0.0081
Catalyst 5	2.0 ± 0.2	0.0141 ± 0.0009	0.1998 ± 0.0214	0.0149 ± 0.0022	0.1954 ± 0.0201
